# Quantitative Modeling of Currents from a Voltage Gated Ion Channel Undergoing Fast Inactivation

**DOI:** 10.1371/journal.pone.0003342

**Published:** 2008-10-03

**Authors:** Carlos J. Camacho

**Affiliations:** Department of Computational Biology, University of Pittsburgh, Pittsburgh, Pennsylvania, United States of America; Boston University, United States of America

## Abstract

Ion channels play a central role in setting gradients of ion concentration and electrostatic potentials, which in turn regulate sensory systems and other functions. Based on the structure of the open configuration of the Kv1.2 channel and the suggestion that the two ends of the N-terminal inactivating peptide form a bivalent complex that simultaneously blocks the channel pore and binds to the cytoplasmic T1 domain, we propose a six state kinetic model that for the first time reproduces the kinetics of recovery of the *Drosophila Shaker* over the full range of time scales and hyperpolarization potentials, including tail currents. The model is motivated by a normal mode analysis of the inactivated channel that suggests that a displacement consistent with models of the closed state propagates to the T1 domain via the S1-T1 linker. This motion stretches the bound (inactivating) peptide, hastening the unblocking of the pore. This pulling force is incorporated into the rates of the open to blocked states, capturing the fast recovery phase of the current for repolarization events shorter than 1 ms. If the membrane potential is hyperpolarized, essential dynamics further suggests that the T1 domain returns to a configuration where the peptide is unstretched and the S1-T1 linker is extended. Coupling this novel hyperpolarized substate to the closed, open and blocked pore states is enough to quantitatively estimate the number of open channels as a function of time and membrane potential. A straightforward prediction of the model is that a slow ramping of the potential leads to very small currents.

## Introduction

Shaker K^+^ channels represent an important model system for studying the molecular basis of how inactivation is coupled to activation and recovery [1]. Most of our knowledge of Kv channels comes from voltage clamp studies on the Shaker K^+^ from *Drosophila* and its mammalian homologs [2]. The recent determination of the crystal structure of the mammalian Shaker Kv1.2 channel-β subunit complex by MacKinnon and collaborators [3] provides for the first time the possibility to clarify relationships between ion channel structure, molecular biophysics, and *in vivo* function. One important feature of the Kv1.2 structure is that it includes the intra-cellular domain preceding the trans-membrane (TM) domains. Namely, before the S1 helix from each of the four channel subunits, the N terminal domains come together to form the tetrameric T1 domain that has been shown to mediate voltage sensitivity [4]–[6]. This domain is located directly under the pore entrance and as clearly shown in the structure together with the S1-T1 linker forms the pathways through which not only the flow of K^+^ ions takes place, but also the channel N terminal peptide, which function as an inactivation mechanism (referred to as “N-type”) in some Shaker family channels [3], can easily fit in.

Fast N-type inactivation was first described in the context of Na^+^ channels by Armstrong and Bezanilla [7] “ball and chain” model. According to this model, inactivation occurs when an N-terminal domain of the α-or-β subunit binds at the pore, blocking the open state of the channel. This mechanism has been amply validated by measurements of ion currents with and without the inactivating peptide (see, also, [8]). Detailed kinetic experiments have further revealed a rich kinetic behavior of macroscopic ion currents as a result of fast inactivation. In particular, Kuo [14] has shown that K^+^ currents recovery from inactivation begins with no delay on repolarization, while hyperpolarization expedite the initial phase of recovery from inactivation yet retard the later phases (see below).

Sequence and mutagenesis experiments have demonstrated the amphiphilic character of this peptide that consists in a hydrophobic “ball” and hydrophilic “tail” [8]–[10]. Long et al. [3] Shaker crystal structure all but confirmed this long accepted mechanism by identifying the hydrophobic region at the channel pore and a tri-peptide motif (E128, D129, and E130) near the S1-T1 linker as possible substrates for the hydrophobic ball and polar tail peptide, respectively.

A quantitative model of ionic currents is crucial for a detailed modeling of the action potential and the mechanisms that it regulates. The kinetics of recovery from inactivation has been extensively studied [1], [8], [10]–[13]. However, so far, there is no model able to provide a detailed quantitative description of ionic currents over the full range of times scales, i.e., from the sub-millisecond to 100 milliseconds range (see, e.g., [14]). For instance, Roux et al. [1] have proposed a 12 state model that incorporates the key notion of parallel pathways, accounting for “fast” and slow phases of recovery but for time scales larger than 1 millisecond. One should mention that most of the states in this model do not have a clear physical origin, and all the parameters of the model are fitted to the experimental data. Indeed, close inspection of these models indicate that the large number of parameters are a direct consequence of the rather strong assumption that the rates dependence on the voltage is always exponential. This assumption is borne out of the expectation that the transition state barrier is proportional to the membrane potential, i.e., similar to the transition between the open and closed channel (see, e.g., [8]). However, if inactivation involves domains outside the membrane, then there is no reason to expect that these states should have the same functional dependence since voltage effects (if any) would be propagated indirectly by electromechanical couplings.

**Figure 1:**
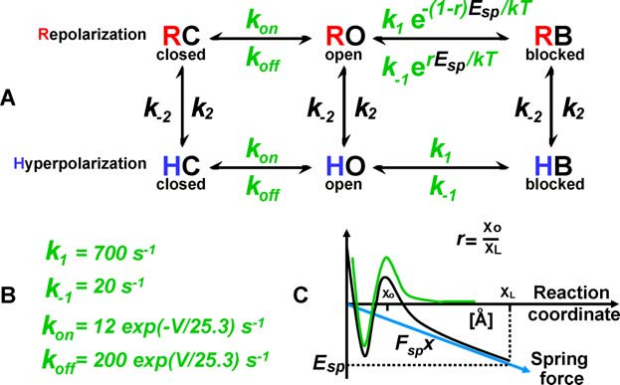
(A) Kinetic model of inactivation and recovery of K^+^ channel. Three pore states: C→closed, O→open, and B→blocked by the inactivating peptide, and two channel substates: R→repolarization (membrane potential less than resting potential), and H→hyperpolarization. The kinetics of recovery is fully determined by three voltage dependent free parameters: a voltage-dependent force of energy *E_sp_* applied to the N-terminal peptide, and the on and off rates *k_±2_* between the R and H substates. (B) Rates obtained from the literature [8]. (C) The free energy landscapes of the peptide blocking the pore in the depolarized state (green) and during repolarization (black). During repolarization, the peptide is assumed to stretch, exerting a force that contributes to the release of the ball from the pore. The dimensions of the ball and the transition state of the mostly hydrophobic ball-pore complex are estimated to be about *X_L_ = 13 Å* and *X_o_ = 3 Å* (∼water layer), respectively (see Methods).

In this paper, we model the kinetics of recovery of a Shaker channel that undergoes N-type inactivation. The model is based on three channel pore states (i.e., open, closed and blocked) and two substates (repolarization and hyperpolarization). The kinetic scheme, shown in Fig. 1A, is perhaps the simplest model yet proposed to account for recovery currents under different conditions. Motivated by a normal mode analysis of the Kv1.2 structure, the model assumes that during repolarization a voltage dependent elastic force pulls the inactivating peptide from the pore, whereas for hyperpolarization potentials there is no pulling force. The analysis suggests that structural transitions are triggered by voltage gating. In particular, a rotation of the S1 helix observed in models of the closed state [15], [16] acts as a “lever” that pushes the S1-T1 linker and T1 domain, generating a relative displacement of the TM and T1 domains that pulls the bivalent complex of the inactivating peptide away from the pore. If the S1-lever rotates too much, the channel can transition back into a state where the peptide is no longer stretched but the S1-T1 linker extends into a new conformation. In combination with Murrell-Lagnado and Aldrich kinetic parameters [8] of N-type inactivation and deactivation (Fig. 1B), the quantitative model resulting from fitting three voltage-dependent free parameters (*E_sp_*, *k_2_* and *k_−2_*) reproduces key experimental observations. For instance, recovery from inactivation [14] is modeled over the full range of hyperpolarization potentials and time scales, including the fast and slow recovery phases for hyperpolarization potentials; and, the time course of tail currents [14], [17]. A striking prediction of our kinetic model is that a slow enough ramping of the membrane potential significantly hinders the ion current [12].

## Methods

### Channel structure

Given the high homology between Kv1.2 and Kv1.4, including *all* relevant domains mentioned here, we use the structure of the former [3] and the Kvβ3 N-terminal as a structural framework of a N-type inactivation channel. The structure of Kv1.2 [3] and Yarov-Yarovoy model of the open channel was further refined to include the S1-T1 linker residues (G131-G160). The linker was mapped into the helix backbone of the crystal and constrained molecular dynamics [18] (MD) were used to relax the helix-helix jointures. We note that S1 and the S1-T1 linker are not fully resolved in the crystal structure, which is consistent with the large fluctuations described here. The resulting model fits well into the crystal template. The above notwithstanding, the precise mapping and side chain models play little or no role in our conclusions. In fact, any level of gating motion on the TM domain will be able to transmit forces to the T1 domain via the S1-T1 linker that regardless of its structure stores entropy.

### Inactivating peptide

The 25-residue long stretch of the N-terminal peptide of Kvβ3 (*M_1_QVSIACTE_9_* **QNL** *R_13_SR_15_SSEDR_20_LCGPR_25_*) was modeled using a 10 ns unconstrained MD in a box with a 15 Å water buffer, resulting in well defined structural motifs. Namely, as shown in the models below, the “ball” end is found to form a tight loop stabilized by a Hydrogen bond between Met1 and Glu9, whereas the more flexible hydrophilic, Arg-rich, tail (residues 13-to-20) clusters around a turn between Ser16 and Asp19-Arg20 that also attracts Arg13. The size of the “ball” extends about 13 Å, i.e., the backbone distance between Glu9.C and Ser4.C, whereas the chain connecting the domain bound to the T1 domain and the ball blocking the pore extends three residues QNL (∼6 Å).

### Blocked state

The “ball” end was first docked to the pore using well validated docking technologies developed in our lab [19], [20], while the tail contacted the T1 domain (as suggested in [3]). The tail complex bound to T1 was further refined using small backbone changes around the main cluster obtained in the MD above. Free energies are estimated based on a scoring function that accounts for electrostatic and desolvation terms [21]. Entropic contributions related to the flexibility of the peptide and the unbound state are not accounted for, though experience tell us that the strong affinity resulting from the peptide tail-T1 complex, −41 kcal/mol, should be strong enough to compensate for the configurational entropy loss.

**Elastic modes** are computed using the server elNemo [22], a tool to compute the low frequency normal modes of a protein treated as a coarse-grained elastic network. These modes have been shown to correlate with large conformational changes in many proteins, e.g., membrane channels [23], [24]. Other normal mode servers that consider only C-alpha atoms provided similar models [25], supporting the notion that the details of the structure are not critical for our findings. All the resulting structures were energy minimized with a standard 100 ABNR steps minimization using the software CHARMm [26], and charmm19 parameters.

### Binding landscape

Figure 1C shows the classical binding free energy landscape as a function of a reaction condition that is defined along the pulling direction. The characteristic length scales of the landscape *X_L_* and *X_o_*, size of the peptide motif blocking the pore and locus of (single) transition state with respect to bound state, are assumed to be about *X_L_ = 13 *Å (size of the “ball”; see above) and *X_o_ = 3 *Å (or about one water layer). These (voltage independent) natural length scales agree well with the predictions of the model, as well with similar models of molecular bonds under an external force (see, e.g., reviews [27], [28]). The pulling force is assumed to be a constant (linear as a function of the pulling distance), which if added to the equilibrium landscape translates in the tilting of the landscape. From the point of view of the kinetics, this force simply changes the height of the transition state, leading to a faster *off* rate and a slower *on* rate (as shown in Fig. 1A).

## Results

### Kinetics of activation, inactivation and recovery of ionic currents on the Drosophila Shaker channel

We propose a simple kinetic model to describe the different states of the voltage-dependent ion channels (Fig. 1). The model recognizes three pore states: (C) closed, (O) open, or (B) blocked by the inactivating N-terminal peptide; and, a repolarization (R) and hyperpolarization (H) substates of the channel. The actual distinction between these two states will become apparent below. These six states correspond to unique conformations, the transition between substates R and H (Fig. 1C) entails a voltage-dependent ensemble of microstates/conformations with varying differences.

### *In silico* modeling of recovery after repolarization

**Figure 2:**
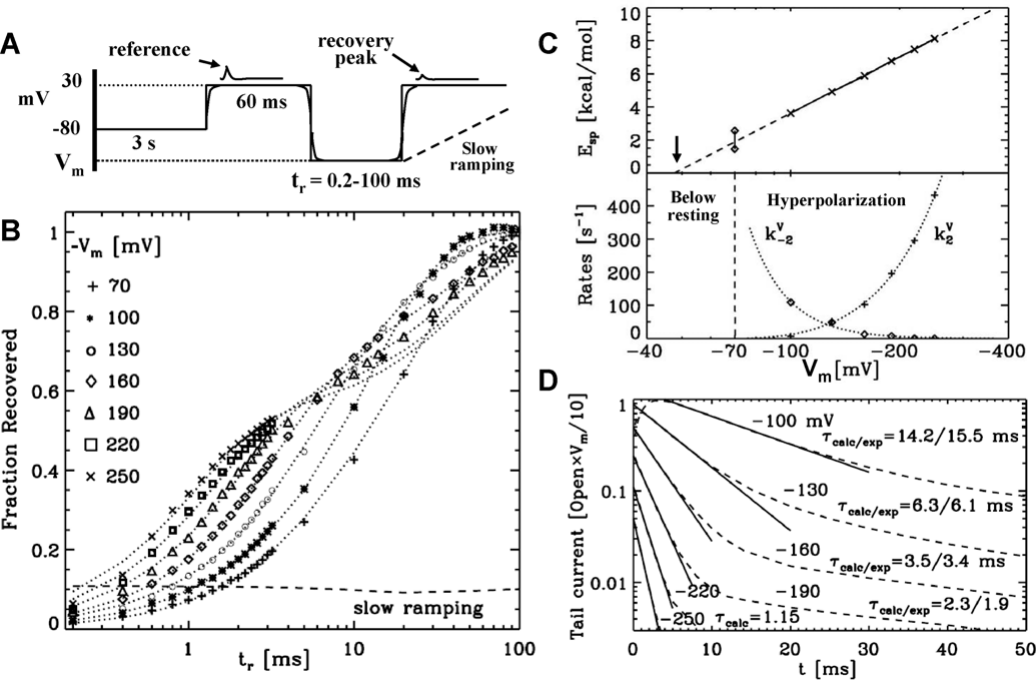
Time course of recovery of a whole-cell *Drosophila* Shaker K^+^ channel. (A) Experimental conditions are taken form Ref. [14]. Oocyte was equilibrated for *3 s* at *−80 mV* in *150 mM* external K^+^, and pulsed twice to *+30 mV* for *60 ms.* The intervening gap between the two pulses is set at various repolarization membrane potentials *V_m_* between *−70 to −250 mV* for a time *t_r_*, which is lengthened by 0.2 ms. A slow ramping potential is also sketched. The currents in the second pulse are used as a measure of the extent of recovery from inactivation relative to the first pulse. The model assumes voltage reversal to be instantaneous. To account for this artifact, peak currents are calculated *1 ms* after depolarization. (B) Experimental (symbols [14]) and predicted (dotted lines) fraction recovered of ion currents as a function of *t_r_*. The fraction is defined as the ratio of the maximum current from the second and first pulse after subtracting the background corresponding to the current at the end of the reference peak. Predictions follow from solving the kinetic scheme in Fig. 1, and assuming that the currents at *V_m_ = +30 mV* are proportional to the concentration of open channels. (C) Model parameters: (Top panel) Elastic energy *E_sp_* of the peptide linker as a function of *V_m_*, arrow points to the threshold voltage below which the blocking rates are predicted to be voltage independent (as in Fig. 1B); Bottom panel: on and off rates *k_±2_* between repolarization (R) and hyperpolarization (H) substates, dotted lines are a guide to the eye. (D) Inward tail currents (dashed lines). For clarity, currents are plotted as the concentration of open channels (RO+HO) times their corresponding voltage. The data is taken from the simulations resulting in Fig. 2B (right at the onset of the second pulse). Solid lines are linear fits used to calculate the time constants and comparison with experiments is noted [14]. For consistency, we disregarded minor differences with the experiment. Namely, in the experiment the first pulse was set to *+60 mV* for *30 ms*, instead of *+30 mV* and *60 ms* in Fig. 1A.

To test our model, we recreate the experimental conditions described in Fig. 2A [14]. As shown in Fig. 2B, the model reproduces in great detail the observed *fraction recovered* of ionic currents over the full range of time scales and repolarization potentials *V_m_≤−70 mV*, where the ionic current is assumed to be equal to the number of open channels at any given time. Upon depolarization the ball rapidly blocks the channels with a constant rate *k_1_ = 700* s^−1^. Repolarization triggers a voltage dependent elastic energy *E_sp_* that is obtained by fitting the experimental data shown in Fig. 2C that pulls the ball from the pore, modifying the binding free energy as shown in Fig. 1C. This parameter controls the initial fast recovery regime observed for repolarization times (*t_r_*) shorter than *1 ms*. An interesting observation is that the fit of the elastic energy *E_sp_* shows a linear dependence on the *log(V_m_)*, which translates into open-to-blocked rates that are linear (not exponential) in the voltage. For membrane potentials below resting (*V_m_<−70 mV; hyperpolarization regime*) the fitted parameters *k_2_* and *k_−2_* suggest a transition to a new substate that no longer stretches the inactivating peptide. The transition to this hyperpolarized substate is voltage dependent, and the on and off rates *k_±2_* are shown in Fig. 2C.

### Modeling a slow ramping of the depolarization potential

Extrapolating the pulling force to zero voltage suggests that the onset of this elastic force is around *−50* *mV* (see dotted line in Fig. 2C). Above this threshold, the ball is not pulled from the pore, i.e., *E_sp_ = 0* *kcal/mol*, consistent with a constant blocking rate of *k_1_* [29]. We use this extrapolation to model a ramping of the membrane potential that is slow compared to *k_1_*. Figure 2B shows a fraction recovered of only 10% when the potential is increased from *−160 mV* to *30 mV* in *0.1* seconds. Furthermore, since currents are modeled based on the number of open channels and RO+HO (Fig. 1A) that peaks at *V_m_<−15 mV* (and not at the reference potential of *+30 mV*), then the true current is bound to be much smaller than the reported 10%.

Of note, a historic reference on this subject can be found in a few lines of the last of the classics Hodgkin & Huxley (A Quantitative Description 1952, pp: 537–538 [12]): “*It is clear that the model will show ‘accommodation’ … so that an applied cathodal current which rises sufficiently slowly will never evoke a regenerative response from the membrane, and excitation will not occur.*” As far as we know, no other model has predicted this effect.

### Tail currents

The validity of the model is further supported by the time course of the predicted inward tail currents shown in Fig. 2D. For these currents, we simply assume that during hyperpolarization there is a reverse current proportional to the number of open channels. Although the absolute value of the current is meaningless, the time constants and overall shape of their decay are not. Indeed, the predicted and experimentally observed (see Fig. 3 in Ref. [14]) time scales are in good agreement. Moreover, we note that the model also reproduces the subtle shift of the maximum current away from *t = 0* as the membrane potential approaches the resting value, see *V_m_ = 100 mV* in Fig. 2D and a similar experiment in Ref. [17]. We note that there are no extra free parameters involved in these predictions.

### Time scale limitations of ideal kinetic models

The main shortcoming of an ideal kinetic model is that changes in membrane potential are assumed to be instantaneous. This is fine if the membrane potential is below resting because peak currents occur at around *1.5 ms* or later. On the other hand, if the repolarization potential is larger or on the order of the resting potential, the peak current upon recovery rapidly shifts from around *1.5 ms* to *0 ms*. This is due to the fact that if the membrane is not hyperpolarized too many channels are left open, triggering a large peak in the current as soon as the potential is reversed. However, this instantaneous transition is not realistic since there is a finite time for reversing the potential (e.g., in [14], the time delay is somewhere between *0.6-to-1 ms*), limiting the validity of any kinetic model to peak currents that occur at times longer than this artificial constraint imposed by the signal generator of the voltage clamp experiment. Note that forcing the fit of this regime to an ideal kinetic model does not make sense unless one explicitly accounts for this effect.

Here, the time delay is modeled implicitly by assuming that the voltage reversal occurs in *1 ms*, this threshold also resembles a reported late transition to the open channel state limited by a voltage-independent ∼*1 ms* time scale [17]. As shown in Fig. 2B, under this condition the model reproduces the recovery times at *V_m_ = −70 mV* and for times shorter than *2.8 ms* at *V_m_ = −100 ms* where the peak currents become instantaneous. For all other conditions, the model does not use this finite time threshold to measure the peak current. Of note, predictions are not too sensitive to this threshold, delays within *0.8-to-1 ms* resulted in similar results.

### Molecular origin of an elastic force that pulls the ball from the pore

The rapid recovery at short time scales, up to around one millisecond of repolarization in Fig. 2B, is accounted for by the pulling force modeled in Fig. 1C and 2C. The origin of this force can be rationalized based on the Kv1.2 structure. In fact, MacKinnon and collaborators model of the blocked state [3], a bi-valent complex where the pore and the T1 domain simultaneously bind to the inactivating peptide, suggests that any relative displacement of these two domains as a consequence of a change in voltage might pull the peptide from the pore.

**Figure 3:**
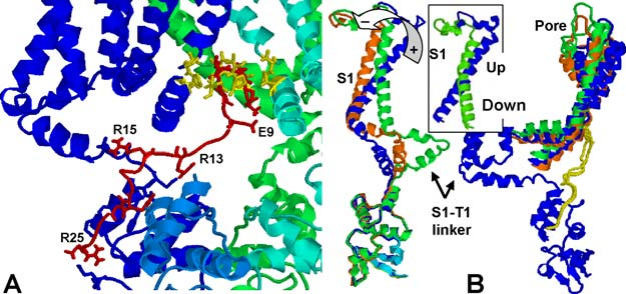
Ball and spring bi-valent complex model of inactivating N-terminal peptide (red) and Shaker Kv1.2 [3]. (A) Shaker includes the S1-T1 (G131-S159) domain modeled here, but has almost no bearing in the complex structure. For clarity, we removed the TM and linker domains of the cyan monomer, Y132-T421. The interaction [21] between the ball (M1-E9) and the pore is dominated by hydrophobic contacts involving residues I5, V3, C7 and M1, and the Valine ring in the pore V406, V410, and P407 (in yellow). On the other hand, the hydrophilic tail binds mostly due to Hydrogen bonds between peptide residues R15, R25, R20, R13 and L21, and T1 domain residues E136, E121, E56, D129, D107 (cyan monomer), E130, M125 and F126, respectively. Chemical affinity (electrostatic plus desolvation) of the ball and tail in the bound state is estimated to be −12 and −41 kcal/mol, respectively [21]. (B) Structural transition upon repolarization modeled using essential dynamics (see Methods). For clarity, only one of the four symmetric monomers is shown: Kv1.2 crystal structure (blue) as in Fig. 3A, repolarized (red) and hyperpolarized (green) models. Arrow indicates direction of S1 rotation due to repolarization. For comparison, inset shows the equivalent view of the S1 helix from the crystal and the “down” model of Yarov-Yarovoy et al. [16]. Also shown are the bound peptides (in yellow) for the crystal and hyperpolarized model structure.

**Figure 4:**
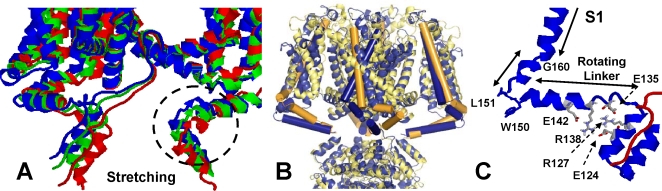
(A) Stretching of inactivating peptide and (B) hyperpolarized state (yellow). For comparison, both models are superimposed at the pore complex with the open crystal structure. (A) Three snapshots are shown, in blue is the starting structure (Fig. 3A and blue chain in 3B) from which we computed the elastic modes in the server elNemo [22], green is an intermediate snapshot and in red is the final state. Sketch shows a 10 Å displacement but we do not have corroborating evidence to estimate an absolute number. For clarity, only two consecutive monomers of the channel and the bound peptides are shown. The dashed circles indicate the helix-turn-helix motif that widens during stretching. The final state in (A)-red (also shown in green in Fig. 2B) is the starting structure used in to model the hyperpolarized state (red chain in Fig. 3B). (B) Note that the T1 domain and pore (see also Fig. 3B) superimpose quite well. Both S1 and the S1-T1 linker are shown as cylinders to emphasize the rotation of S1 and the ratchet motion of the linker that is in a more extended conformation (see, also, Fig. 3B-green model). (C) Detailed of the S1-T1 linker and T1 domain interactions (as in Fig. 3A). Some key residues on the linker-T1 interface are shown. Note that E142 forms two H bonds with R138 which forms one H bond with T1 E124. The second H bond of E124 is with R127 that is also bonded to E135 in the linker.

The possibility of a relative displacement of the TM and T1 domain is further supported by a normal mode analysis applied to a structural model (as in Ref. [3]) of the blocked. We first note that both Yarov-Yarovoy et al. [16] and Grabe et al. [15] model of the down state translate in a counter-clock rotation of the outer TM helix S1. Using this rotation as a probe for the modes that might be sampled by voltage gating, we identified two modes (among the 30 slowest normal modes) that are consistent with the overall displacement of the S1 helix. In these two modes all four tetrameric domains move in synchrony and the outer S1 helix rotates counter-clockwise with respect to the pore (see inset in Fig. 3B for a comparison of the rotation of S1 between the open and closed state in [16] with respect to the one observed here). Mode 1 tilts S1, pushing the S1-T1 linker, which instead pushes T1 away from the TM domain (Fig. 4A). On the other hand, mode 2 pushes T1 sideways leading almost immediately to clashes, as the linker and T1 interface collapse with each other. A straightforward 4x30 steps energy minimization using CHARMm [26] confirms that the internal and van der Waals energies rapidly increase along mode 2, but not mode 1. Hence, the prediction is that repolarization triggers a structural rearrangement consistent with mode 1, which as shown in Fig. 4A pulls the bound peptide from the pore. This coupling between TM and T1 in the blocked state is consistent with an elastic force like the one modeled in Fig. 1C.

### Hyperpolarization regime

It is important to emphasize that the structural rearrangements of the channel are not due to thermal fluctuations. Instead, they are driven by voltage gating, i.e., the rotation of S1 that is covalently linked to the cytoplasmic domain. This transduction of voltage to mechanics relates to the rotation of the S1 domain that seems to work as a molecular “lever.” If this lever keeps pushing, the S1-T1 linker eventually rotates as in the normal mode 2 described above, since the helix-turn-helix motif highlighted in Figs. 4A and 4C has widened during repolarization, removing the aforementioned clashes between the linker and T1 domain. This dynamical transition is modeled by performing a new normal mode analysis from the stretched conformation in Fig. 4A. In this case, we observe that the S1-T1 linker reaches a more extended conformation (see green chain in Fig. 3B and Fig. 4B), while T1 retracts back into its original conformation relative to the pore domain. The final state differs from the original crystal in that the voltage sensor is in the “off” state and, the rotating stretch of the S1-T1 helical linker, Gly131-Trp150 (see Figs. 4B and 4C), has ratcheted forward by roughly 23°. Of note, the extension of the S1-T1 linker observed in Fig. 4B is fully consistent with the latest structure of this domain [30], where it is said that this domain is “probably extended over part of its length.” Moreover, the fact that we recover a repolarized structure roughly similar to Catterall's model from a completely independent analysis of the Kv1.2 structure provides added support to our analysis. The above notwithstanding, we should emphasize that these structural transitions are only models allowed by the molecular mechanics of the structure, and motivated by the slowest elastic normal models.

As shown in Fig. 4B, the hyperpolarized conformation brings back the TM and T1 domains to a conformation similar to that of the original “open” crystal structure. This observation is the main motivation to postulate the hyperpolarized substate in Fig. 1, i.e., at very low membrane potentials the (fast) blocking and (slow) unblocking rates are the same as in the depolarized state (*E_s_ = 0*), slowing down recovery. On the other hand, above the membrane resting potential inactivation remains voltage-dependent.

## Discussion

The 6 state kinetic model presented in Figs. 1 and 2 fully accounts for experimental observations that have never been modeled over the full range of experimentally resolved time scales and with half the states of previous models (a direct comparison with the state of the art model can be found in the supplemental Fig. S1). We note that, in the past, a single recovery curve required at least three exponentials (6 parameters) in order to fit the different regimes in Fig. 2B. The kinetic scheme in Fig. 1A requires just three parameters, the voltage dependent elastic energy *E_sp_* and the rates *k_±2_* in Fig. 2C. The model also assumes a binding landscape for the unstretched peptide and a time delay for the reversal of the voltage. Together with experimentally validated kinetic estimates [8], the model accounts for the detailed kinetics of activation, inactivation, recovery and inward tail currents, as measured by voltage clamp experiments [14].

For *V_m_*∼−*70 mV* and above, the model is highly sensitive to fast time scales. The reason for this is straightforward, i.e., after repolarization too many channels are left open and the peak current occurs *immediately* after depolarization. This is an artifact since the time scale to reverse the potential between polarized and depolarized conditions is finite (∼*1 ms*). It is clear that no model that assumes an instantaneous reversal of the potential should fit the data. Instead, the model should carefully model the time dependence of the voltage generator. Here, we solve this issue by simply limiting the peak current to at least *1 ms* after depolarization. One might also be able to force the model to predict the currents, and in such cases extra parameters are needed. For instance, we built a second model for *V_m_ = −70 mV*, where we assumed a recoil to an intermediate state that increases *E_sp_* by an extra *1.4* *kcal/mol*, as opposed to retracting to the original stretching of the depolarized state with *E_sp_ = 0*. This second model shifts the current peak to around *1 ms* after depolarization, appropriately fitting the data. Ultimately, better time resolution of the voltage is needed to fully resolve this issue.

Assuming a reverse or inward “tail” current proportional to the number of open channels during hyperpolarization is enough to predict “tail” currents that are in full agreement with experimental measurements. We note that Hodgkin and Huxley model [31] suggested that tail currents were the result of channels that remained open at the end of the first pulse, which is actually the case for *V_m_<−130 mV*. However, for *V_m_>−130 mV*, tail currents are mostly due to channels that were first inactivated and reopened in their way to the closed state, as first suggested by Demo and Yellen [11].

A striking prediction of the model is that if we assume that the scaling of the parameters described in Fig. 2C are appropriate for any voltage, then ramping the membrane potential at a rate slower than *k_1_* leads to insignificant ionic currents. The reason for this is that, for *V_m_>−50 mV*, the N-terminal peptide will block the ion channel before reaching the fully depolarized state. Hence, ionic currents are predicted to strike only if the reversal of the membrane potential is faster than the blocking rate. Interestingly, this phenomenon was apparently last quantitatively described by Hodgkin and Huxley [12].

A unique feature of our model is that we show that each of the six states is consistent with the structural constraints of the Shaker channel. In fact, the states correspond to the classical open, closed and blocked state, where each of these states can be in either a (re)polarized or hyperpolarized substate. Collectively, this study provides insights pertinent to a new level of understanding of the kinetic coupling of ionic currents. In agreement with experiments, we find (a) a late stage voltage-independent rate of channel closing [32], whose limiting step during hyperpolarization is *k_on_*, i.e., the transition between HB→HO where *k*_−1_<<*k_on_*; (b) the molecular mechanism of the slow inward tail current; (c) without any sophisticated modeling technique, ionic currents have probed the validity of the model of the “down” state that rotates the S1 TM domain, see Caterall's and Jan's models [15], [16]; (d) finally, the role of the S1-T1 linker as a key regulator of Kv-like channels is further supported by its conservation across distant species (e.g., 90% conserved between Drosophila and Human), as well as the recently reported [30] extended state of this domain (see model in Figs. 3B and 4B). The latter is also consistent with recent mutation studies in Kv channels [4], [33], as well as in Ca^+2^ activated channels [34] where its linker was also shown to regulate ionic currents.

Our findings present a comprehensive view of the kinetic transitions responsible for the regulation of ionic currents in Shaker K^+^, suggesting that S1 and the S1-T1 linker play a critical role communicating voltage stimuli to the intracellular domains. In the presence of N-type inactivation, we show that this stimuli couples to the kinetics of inactivation and recovery. The structural models suggest that a similar mechanism might apply for channels without a “ball and chain” mechanism since the ratchet motion of the S1-T1 linker in Fig. 4B is independent of the N-terminal peptide. Further analyses are required to confirm these hypotheses. Nevertheless, the combination of electrophysiological and structural experiments has proven useful to develop a quantitative model of ionic currents for the full range of experimental available time scales, from sub-millisecond to *100 ms*.

## Supporting Information

Figure S1 Direct comparison to state of the art quantitative model of ion currents
(0.12 MB DOC)
