# Supplementary material for: Quantitative Modeling of Currents from a Voltage Gated Ion Channel Undergoing Fast Inactivation
Source: PLoS One. 2008 Oct 3;3(10):e3342. doi: 10.1371/journal.pone.0003342 (PMC2551740; doi:10.1371/journal.pone.0003342)
Supplement: Figure S1 — Direct comparison to state of the art quantitative model of ion currents (0.12 MB DOC) [file pone.0003342.s001.doc]

| **Figure S1. Direct comparison to state of the art quantitative model of ion currents** | |
| --- | --- |
| **PLoS One model** | **Roux, Olcese, Toro, Bezanilla, Stefani (Ref. [1])** |
| **6 states and 7 rates (Fig. 1).** | **12 states and 24 rates (Fig. 13)** |
| Four of the parameters are experimentally determined (green); 3 are fitted to the data **E_sp_, k_2_ and k_-2_.** | All 24 parameters are fitted to the data. |
| Fitting of the data is excellent in the range between 0.1 ms to 100 ms for 7 potentials (Fig. 2B). | Fitting of the data is fine in a *linear* scale between 50-to-100 ms for 4 potentials (Fig 15C). |
| **Predict** inward tail currents (Fig. 2D). | **No quantitative model has ever predicted both outward and inward currents.** |
| **Predict** decrease of currents with slow ramping of polarization (Fig. 2B). | The last model to comprehensible model this phenomena was the classic Hodgkin & Huxley (A Quantitative Description…1952 pp:537-538): “*It is clear that the model WILL show “accommodation” … so that an applied cathodal current which rises sufficiently slowly will never evoke a regenerative response from the membrane, and excitation will not occur.*” |
| **Physically motivated in Shaker structure and model from R. MacKinnon (Science 2005).** | **Motivation is fitting the data of the currents responsible for the action potential and the critical functions that they regulate.** |
